# Regulation of Angiopoietin Signalling by Soluble Tie2 Ectodomain and Engineered Ligand Trap

**DOI:** 10.1038/s41598-017-03981-6

**Published:** 2017-06-16

**Authors:** Deborah O. A. Alawo, Tariq A. Tahir, Marlies Fischer, Declan G. Bates, Svetlana R. Amirova, Nicholas P. J. Brindle

**Affiliations:** 1 University of Leicester Department of Cardiovascular Science and Department of Molecular and Cell Biology, Henry Wellcome Building, Lancaster Road, Leicester, LE1 7RH UK; 2 University of Warwick, Department of Engineering, School of Engineering, Coventry, CV4 7AL UK; 3 Advanced Data Mining International LLC, 3620 Pelham Road, PMB 351, Greenville, SC 29615 USA

## Abstract

Angiopoietin-1 (Angpt1) is a glycoprotein ligand important for maintaining the vascular system. It signals via a receptor tyrosine kinase expressed on the surface on endothelial cells, Tie2. This receptor can undergo regulated ectodomain cleavage that releases the ligand-binding domain (sTie2) into the circulation. The concentration of sTie2 is increased in a range of conditions, including peripheral arterial disease and myocardial infarction, where it has been suggested to bind and block Angpt1 resulting in vascular dysfunction. Here we use a joint mathematical modelling and experimental approach to assess the potential impact of sTie2 on the ability of Angpt1 to signal. We find that the concentrations of sTie2 relative to Angpt1 required to suppress signalling by the ligand are more than ten–fold higher than those ever seen in normal or disease conditions. In contrast to the endogenous sTie2, an engineered form of sTie2, which presents dimeric ligand binding sites, inhibits Angpt1 signalling at seventy-fold lower concentrations. While loss of Tie2 ectodomain can suppress Angpt1 signalling locally in the cells in which the receptor is lost, our study shows that the resulting increase in circulating sTie2 is unlikely to affect Angpt1 activity elsewhere in the body.

## Introduction

The angiopoietins are a group of secreted glycoprotein ligands that act mainly on the vasculature^1^. In humans there are three angiopoietins, angiopoietin-1 (Angpt1), Angpt2 and Angpt4^2–4^. Angpt1 is constitutively secreted under normal conditions by perivascular cells^4^ and has crucial vascular protective effects, acting to maintain endothelial quiescence, suppress vessel inflammation, prevent microvessel regression and inhibit vascular leakage^5^.

The primary receptor for angiopoietins is the receptor tyrosine kinase Tie2^2–4^. This transmembrane protein is expressed on endothelial cells^6, 7^. Tie2 is activated by Angpt1, stimulating receptor phosphorylation, intracellular signalling and functional effects^1, 5^. Endothelial cell effects of Angpt1 include suppression of apoptosis, enhancement of cell:cell junction integrity and inhibiting expression of inflammation-associated genes^1, 5^.

In addition to cellular Tie2, a soluble form of the angiopoietin receptor has been detected in the circulation^8, 9^. Soluble Tie2 (sTie2) originates from endothelial cells where it is generated by proteolytic cleavage and release of the ectodomain from full-length receptor located at the cell surface^8, 9^. sTie2 contains the ligand binding domain of the receptor and can bind angiopoietins, preventing them from interacting with cellular Tie2 and thereby inhibiting angiopoietin action^9^. sTie2 is increased in a number of diseases^10–14^. The possibility that increased sTie2 could contribute to vascular pathology by inhibiting Angpt1 protective signalling has been highlighted in peripheral arterial disease, myocardial infarction, vessel permeability in inflammation, organ failure in post cardiac-arrest syndrome and trauma, pulmonary hypertension, and neoangiogenesis^8, 12, 15–19^. sTie2 therefore may be an important target for therapeutic inhibition.

sTie2 has the potential to have profound effects on regulating angiopoietin availability and contribute to vascular pathology in diseases in which it is elevated. Despite this we know relatively little about how sTie2 affects the ability of angiopoietins to act on endothelial cells. In this study, therefore, we use computational modelling and experiment to assess the effects of sTie2 on the ability of Angpt1 to bind and activate its cellular receptor.

## Results

**Figure 1:**
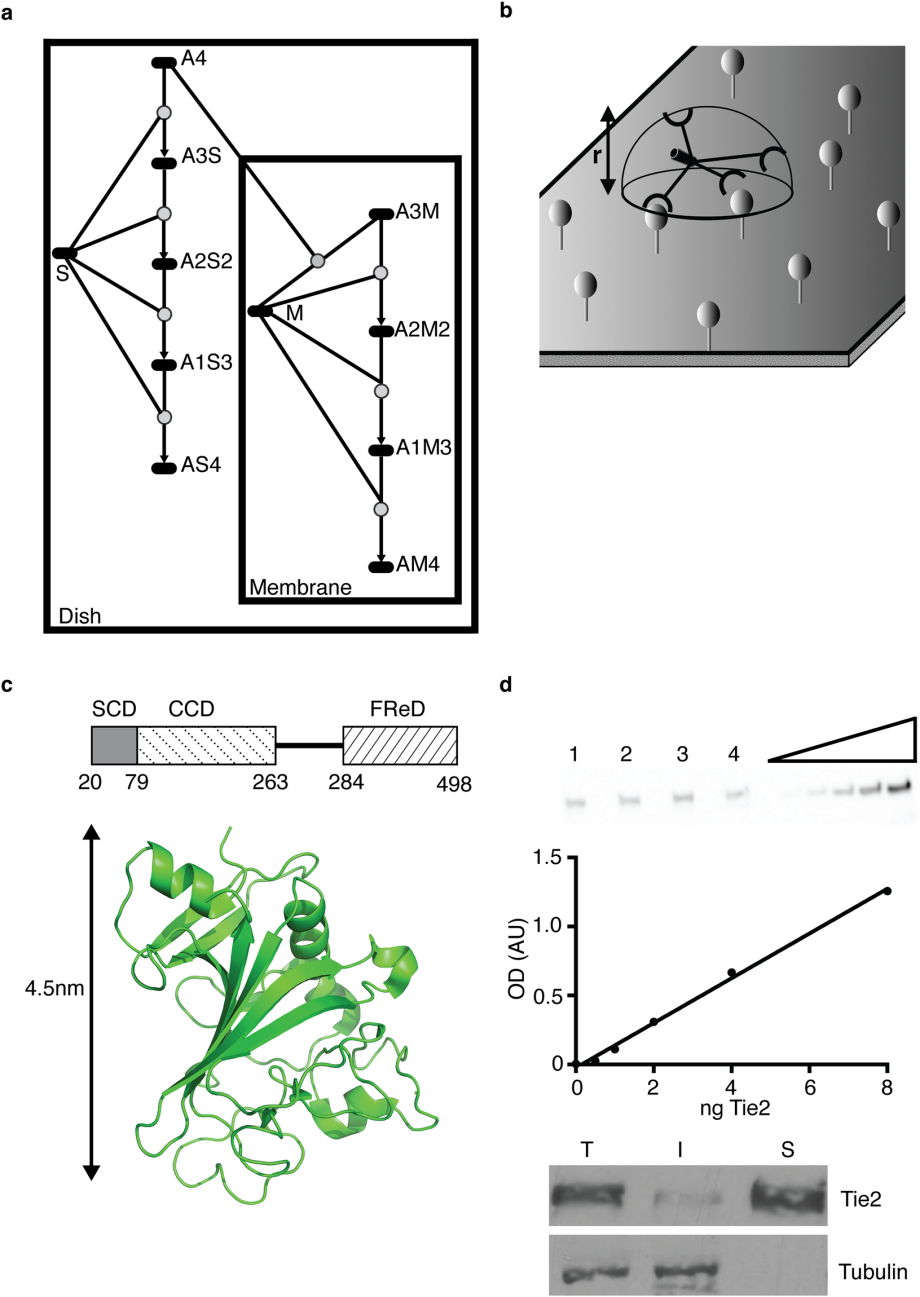
Mathematical model for kinetics of Ang1 binding to Tie2. (**a**) Schematic diagram of Ang1 interactions represented in Simbiology Toolbox, Matlab. A two-compartment model was constructed with Ang1 binding to sTie2 and the initial recruitment of Ang1 to cellular Tie2 occurring in the extracellular compartment. Recruitment of additional cellular Tie2 receptors occurs in the membrane compartment. Ang1 is represented by A, sTie2 by S, and cellular Tie2 by M. The suffix following A indicates the available receptor binding sites and the suffix following M and S the number of receptors bound to each tetrameric ligand. (**b**) Representation of the space in which Ang1 bound to a cell surface receptor can search for further membrane receptors. The distance above the membrane that the ligand can search is designated by r and represents the distance between binding sites on maximally extended Ang1. (**c**) Schematic representation of Ang1 showing super-clustering (SCD), coiled-coil (CCD) and fibrinogen related domain (FReD) of a monomer together with amino acid numbers at domain junctions, secretory leader domain (residues 1–19) not shown. Below: Structure of Ang1 fibrinogen related domain^39^ that contains the C-terminal receptor binding domain is shown together with length indicator. Structure visualized with PYMol. (**d**) Estimation of Tie2 number per cell. Upper blot shows an example of an anti-Tie2 immunoblot used for quantification. Numbers 1–4 designate four different cell lysates and tracks to the right of these are known amounts of recombinant Tie2. Below the blot is an example of a standard curve derived from the blot and used to calculate total Tie2 content of HUVECs. Beneath the standard curve is an example of a blot to determine percentage distribution of cellular Tie2 exposed on the cell surface. Surface Tie2 was labelled with cell impermeant NHS-LC-biotin and biotinylated surface receptor was recovered with streptavidin-agarose. Aliquots of total (T), intracellular (I) and cell surface (S) fractions were resolved by SDS-PAGE and probed for Tie2 or the intracellular protein tubulin, by immunoblotting, as indicated. Images of blots have been cropped for clarity.

In order to understand the influence sTie2 could exert on the ability of angiopoietins to bind cell surface Tie2 we constructed a simple mathematical model based on binding kinetics^20^ for Angpt1 binding in the absence and presence of sTie2 (Fig. 1a and Supplementary Information). Angpt1 is a multimeric ligand existing in trimeric, tetrameric and pentameric structures as well as higher order aggregates^21^. We examined the case for tetrameric Angpt1, as this is the lowest order oligomeric form of the ligand able to activate Tie2 in endothelial cells^21, 22^. Tetrameric Angpt1 is able to form complexes with up to four cell membrane localized Tie2 receptors. These complexes are represented by A3M, A2M2, A1M3 and AM4 in our model, where the suffix following A indicates the available receptor binding sites on Angpt1 and the suffix following M the number of membrane receptors bound to each tetrameric ligand. Similarly, binding of Angpt1 to soluble Tie2, designated S in the model, can give rise to A3S, A2S2, A1S3 and AS4_._

sTie2 binding to Angpt1 occurs in the extracellular space volume. Similarly, the first interaction of Angpt1 with the cell surface receptors occurs by recruitment of Angpt1 from the extracellular medium space. However, once the ligand binds to a cell surface Tie2, subsequent interaction with further membrane Tie2 receptors occurs in a much more restricted volume at the cell surface. A two-compartment model was therefore constructed representing the extracellular space and cell surface compartment, respectively (Fig. 1a). As illustrated in Fig. 1b, the reaction volume at the cell surface corresponds to the cell surface area and height above the surface that the ligand can sample. Assuming Angpt1 is completely flexible, once initially recruited to a cell surface receptor the remaining free binding sites on the tetrameric ligand are able to sample a radius of up to 25 nm above the cell surface (Fig. 1c and Supplementary Information). The decrease in search volume at the surface, compared with the volume of the extracellular space, substantially increases the likelihood of interaction between surface-localized ligand:receptor complexes and vacant cell surface receptors. In order to model Angpt1 binding to cellular Tie2 it was necessary to determine the number of cell surface Tie2 receptors. This was done by immunoblotting endothelial cell lysates and comparison with standard curves of Tie2, which estimates 1.8 × 10^5^Tie2/cell (Fig. 1d and Supplementary Information). Using surface biotinylation 86.9% of these receptors were localized to the cell surface (Fig. 1d and Supplementary Information). Details of the two-compartment model are provided in Supplementary Information.

**Figure 2:**
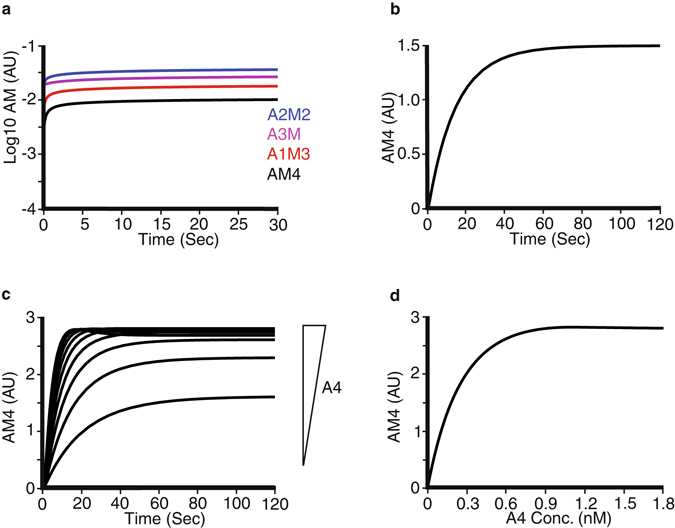
Numerical simulations of Ang1 binding to cellular Tie2. The mathematical model described in Supplementary Information was utilized to examine the time course of formation of cell surface complexes between 0.18 nM Ang1 and cellular Tie2. (**a**) Time course of formation of Ang1 bound to one (A3M; purple), two (A2M2; blue), three (A1M3; red) and four (AM4; black) surface receptors. Amount of complex is shown in log scale (arbitrary units). (**b**) Time course of the formation of Ang1 fully complexed with four cell surface receptors (AM4). (**c**) Time course of formation of AM4 at different Ang1 concentrations up to 1.8 nM. Increasing Ang1 concentration is indicated as an inverted triangle. (**d**) Dependence of AM4 formation on Ang1 (A4) concentration.

In initial numerical simulations we examined the formation of ligand-receptor complexes at the cell-surface in the absence of sTie2 and with 0.18 nM Angpt1. This concentration of Angpt1 was chosen as it has been used in a number of cell culture studies^23–25^ and is a low concentration that would be expected to be more sensitive to sTie2 inhibition than higher Angpt1 concentrations. The predominant type of the ligand:receptor complex formed is A2M2 (Fig. 2a), the ligand complexed with two receptors. This is the complex formed after the initial recruitment of Angpt1 by cell-surface Tie2 to form A3M and thus represents the first entirely membrane compartmentalized interaction, between the membrane localized ligand-receptor complex with vacant membrane receptors. Levels of A3M are lower than A2M2 and lower still are ligand bound to three receptors (A1M3). The least abundant complex is the tetrameric AM4 complex (Fig. 2a), and the time course of AM4 formation is shown in Fig. 2b. The effects of changing Angpt1 concentration on formation of the tetrameric ligand-receptor complex, AM4, was investigated up to 1.8 nM Angpt1 and showed a concentration-dependent increase in complex formation Fig. 2c,d).

**Figure 3:**
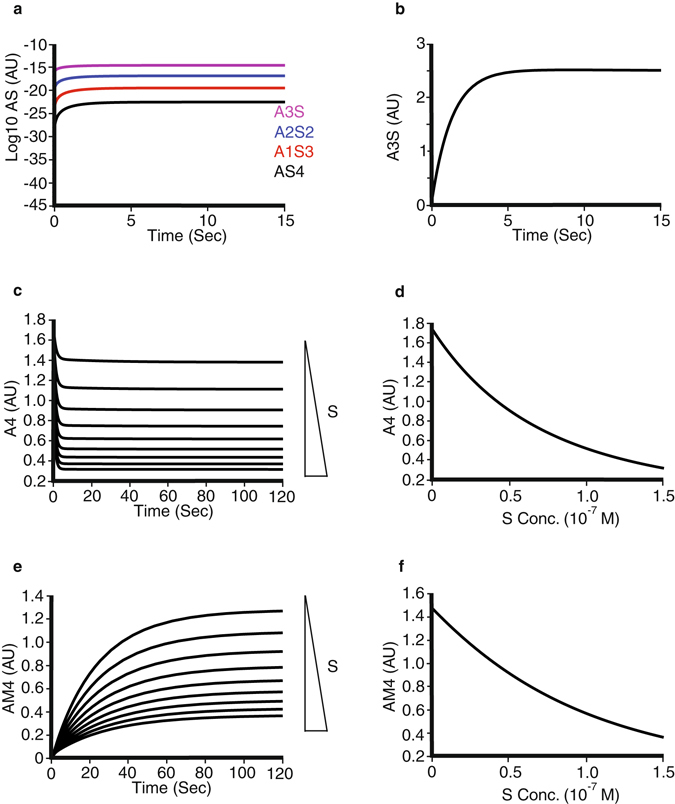
Numerical simulations of the effects of sTie2 on Ang1 binding. (**a**) Time course of formation of Ang1 bound to one (A3S; purple), two (A2S2; blue), three (A1S3; red) and four (AS4; black) soluble Tie2 ectodomains. Initial Ang1 concentration 0.18 nM, initial sTie2 concentration 1 nM. Amount of complex is shown in log scale (arbitrary units). (**b**) Time course of the formation of Ang1 complexed with a single sTie2 (A3S). (**c**) Time course of effects of different sTie2 concentrations up to 150 nM on free Ang1 (A4) concentration starting with a free Ang1 concentration of 0.18 nM (**d**) Concentration-dependence of sTie2 effects on free Ang1 (A4) concentration. (**e**) Time course of the effects of increasing sTie2 concentrations on the ability of 0.18 nM Ang1 to form the signalling-competent AM4 complex at the cell surface. (**f**) Concentration-dependence of sTie2 effects on formation of signalling-competent AM4 complex at the cell surface.

Correspondingly, the effects of adding sTie2 were examined in the model (Fig. 3a). sTie2 was able to bind and complex ligand, and all four species of soluble receptor-ligand complex were observed (Fig. 3a). In contrast to the cell-surface receptor, the major species of ligand complex formed with soluble receptor was Angpt1 bound to a single sTie2 (A3S), and this is formed within seconds of sTie2 addition (Fig. 3b). Model prediction for addition of soluble receptor to Angpt1 (A4) resulted in a drop in the concentration of free ligand and, as expected, this was dependent on the concentration of sTie2 (Fig. 3c,d).

Consistent with its effects on free ligand concentration, the soluble receptor suppressed formation of cellular AM4 complexes (Fig. 3e,f). Surprisingly however, soluble receptor was required in relatively high concentrations to impact on formation of tetrameric complexes of ligand and cell surface Tie2 (Fig. 3e,f). Formation of AM4 in the absence and presence of equimolar soluble receptor were indistinguishable. With 0.18 nM Angpt1, the soluble receptor ectodomain only produced clear effects on membrane receptor complexes when present at 10 nM and above, and around 70 nM sTie2 was required for 50% inhibition of cellular Tie2 tetramerization (Fig. 3e,f). This represents a molar ratio of more than 350 fold sTie2:Angpt1 required to suppress formation of the signalling-competent AM4 complex by 50% (Fig. 3f).

Cellular Tie2 expression is increased at sites of angiogenesis^26^. We therefore examined the effects of increasing cellular Tie2 on the ability of sTie2 to modulate Angpt1-induced receptor tetramerization. Increasing cellular Tie2 levels ten-fold increased the concentration of sTie2 required to suppress Angpt1-induced AM4 formation to 122 nM (Supplementary Fig. S1). For comparison we also examined the effects of decreasing cellular Tie2 levels. A ten-fold decrease in cellular Tie2 caused the concentration of sTie2 required for 50% inhibition of Angpt1-induced Tie2 tetramerization to fall to 57 nM (Supplementary Fig. S1), a molar ratio of still more than 300:1 sTie2:Angpt1.

**Figure 4:**
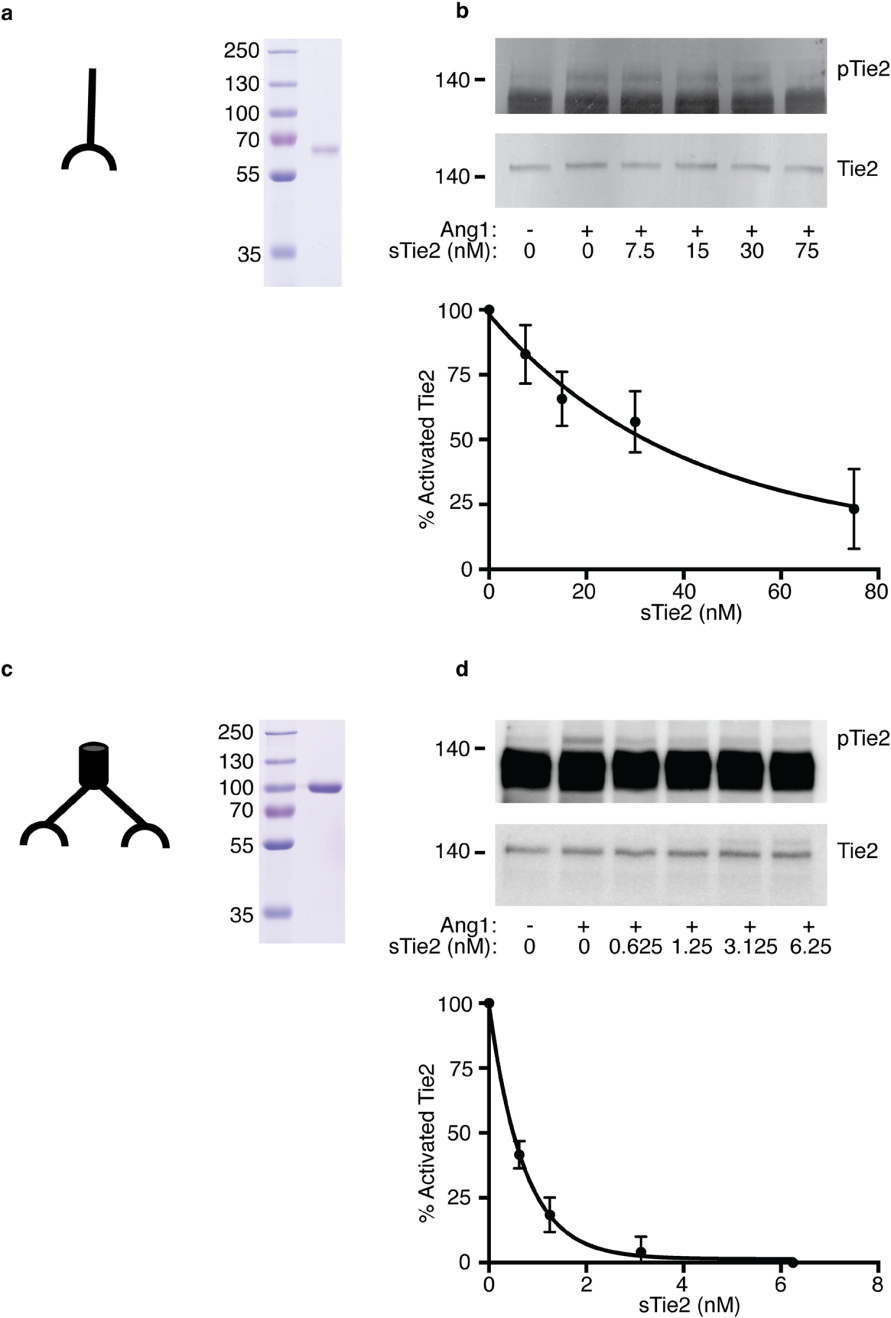
Effects of sTie2 on Ang1 activation of cell surface Tie2. (**a**) Coomassie-stained gel showing purified monomeric sTie2 used in experiments, mass markers are indicated in kDa. (**b**) Effects of sTie2 on Ang1 activation of cellular Tie2. HUVEC were stimulated with 0.18 nM Ang1 for 15 minutes in the presence of monomeric sTie2 concentrations shown. Cells were lysed and activated cellular Tie2 detected with anti-phosphoY^992^-Tie2 immunoblotting. Blots were stripped and probed with anti-Tie2 to quantify loading of tracks. (**c**) Blots from three independent experiments assessing cellular Tie2 activation were quantified by densitometric scanning and presented as mean and SEM for the effects of sTie2 on cellular Tie2 activated with Ang1. (**d**) Coomassie-stained gel showing purified dimeric engineered sTie2 used in experiments, mass markers are indicated in kDa. (**e**) Effects of dimeric sTie2 on Ang1 activation of cellular Tie2. HUVEC were stimulated with 0.18 nM Ang1 for 15 minutes in the presence of dimeric sTie2 concentrations shown. Cells were lysed and activated cellular Tie2 detected with anti-phosphoY^992^-Tie2 immunoblotting. Blots were stripped and probed with anti-Tie2 to quantify loading of tracks. (**f**) Blots from four independent experiments assessing cellular Tie2 activation were quantified by densitometric scanning and presented as mean and SEM for the effects of sTie2 on cellular Tie2 activated by Ang1. Images of blots have been cropped for clarity.

Our numerical simulations predict that high sTie2:Angpt1 molar ratios will be required to inhibit formation of the signalling-active tetrameric Tie2 complex (AM4) on endothelial cells. To test this prediction we directly examined the effects of sTie2 on the ability of Angpt1 to activate cellular Tie2. Human endothelial cells were challenged with Angpt1, or control vehicle, in the absence or presence of sTie2 (Fig. 4a), and receptor activation was monitored by assessing phosphorylation of Y992 in the activation loop of Tie2^27^. Addition of 0.18 nM Angpt1 to cells resulted in activation of Tie2 (Fig. 4b). This activation was inhibited only 10% by a 40-fold molar excess of sTie2 and required 194-fold molar excess of sTie2 for 50% inhibition (Fig. 4b). Addition of sTie2 in the absence of Angpt1 did not activate the receptor (Fig. S2), confirming that any receptor activation seen with Angpt1 in the presence of sTie2 reflects lack of inhibition by sTie2 rather than sTie2-induced receptor activation. These data support the predictions from the model of high molar excesses of sTie2 being required to inhibit Angpt1 action.

Soluble receptor ectodomains are used as therapeutic ligand-traps to block effects of pathological or inappropriately elevated concentrations of ligand^28^. Examples of such ligand-traps include TNF-α receptor ectodomain, Etanercept, used in treatment of rheumatoid arthritis^29^, and the VEGF ligand-trap, Aflibercept, used in a number of pathologies including diabetic macular oedema^30^. In contrast to physiological soluble receptors, ligand-traps are usually fusion proteins in which the ligand-binding domain is engineered to be presented in a dimeric format^28^. We were interested to investigate how a dimeric form of sTie2, similar to that which would be used for a ligand trap, compares with monomeric sTie2 in their ability to affect Angpt1 action. As shown in Fig. 4d, and in contrast to monomeric sTie2 (Fig. 4b), receptor activation was clearly inhibited at much lower concentrations by the dimeric version of sTie2, with 50% inhibition at around 0.5 nM dimeric sTie2 and complete inhibition at around 3 nM.

## Discussion

Our results show that Angpt1 activation of cellular Tie2 is poorly responsive to sTie2. We found that in order to suppress signalling-competent Tie2 complex formation by 50% sTie2 is required at concentrations in the range of 350–200 fold that of Angpt1 in our model and experiments. Concentrations of Angpt1 in humans under normal conditions are around 30–70pM^10, 13, 16, 31^ and vary little with disease, though a decrease to 14pM has been reported in sepsis^17^. Circulating sTie2 concentrations in humans have been reported to be in the region of 100–200 pM in normal conditions^10, 13, 16, 31^, rising as high as 350 pM in severe sepsis^17^. The molar ratio of sTie2:Angpt1 found *in vivo* therefore is normally around 3–6 rising to 25 in severe sepsis. Such relative concentrations are much lower than those we found to affect the ability of Angpt1 to assemble tetrameric Tie2 complexes in our model and suppress Tie2 activation in our experiments. This suggests that circulating sTie2 is unlikely to influence the ability of circulating Angpt1 to activate cellular Tie2 at the concentrations found in normal and disease states *in vivo*.

It is possible that additional factors present *in vivo* and not incorporated in our model and experiments could enhance the ability of sTie2 to modify Angpt1 action, although there is no evidence for such factors to date. In fact, *in vivo* sTie2 is likely to be less effective at suppressing Angpt1 action on endothelium than our data suggest. This is because *in vivo* Angpt1 is released by pericytes and smooth muscle cells on the abluminal surface of the endothelium, and the ligand is also present in the extracellular matrix around vessels^4^. Therefore, to access this pool of Angpt1 circulating sTie2 would have to cross the endothelial permeability barrier to access the ligand. In a number of diseases where sTie2 is increased this is accompanied by an increase in Angpt2^10, 13^. As Angpt2 is able to bind Tie2^3^ it is conceivable that where Angpt2 concentrations are increased sufficiently this ligand could compete with Angpt1 for binding to sTie2 and further decrease the ability of sTie2 to modulate Angpt1.

Cellular levels Tie2 are known to be increased at sites of angiogenesis^26^. There have also been reports of Tie2 levels varying across different vascular beds^32^, although others report uniformity in Tie2 expression^33^. Our simulations show that sTie2 remains relatively ineffective at modulating Angpt1-activation of cellular Tie2 tetramerization even when cellular Tie2 levels are varied over a 100-fold range. Thus we found that increasing cellular Tie2 levels ten-fold further decreased the effectiveness of sTie2 to affect Angpt1 action. Decreasing cellular Tie2 levels in our simulations lowered the concentration of sTie2 required to cause 50% suppression of Angpt1-induced cellular Tie2 activation to 57 nM. Even this lowered concentration of sTie2 corresponds to a molar ratio of more than 300 fold sTie2:Angpt1. Further decreases in cellular Tie2 are likely to increase sensitivity to sTie2, though at such low levels of cellular Tie2 the signal from the cellular receptor will be negligible irrespective of sTie2.

Our data do not discount Tie2 shedding as an important mechanism regulating endothelial responses to Angpt1. Clearly, proteolytic shedding of cell surface Tie2 would suppress the ability of Angpt1 to signal through Tie2 in cells in which shedding has occurred. Furthermore, it is possible that local very high concentrations of released sTie2 could accumulate abluminally or be present near the surface of endothelial cells undergoing proteolytic Tie2 shedding. In both of these situations Angpt1 actions could be inhibited in the locality of the cells undergoing shedding, although the ability of sTie2 to affect Angpt1 even locally could be blunted where Angpt2 is also locally released and able to compete for sTie2 binding. Furthermore, as discussed, the released ectodomain is unlikely to affect circulating or abluminal Angpt1 action elsewhere in the vasculature, minimizing systemic effects. Others have also questioned whether sTie2 has any systemic effect on Angpt1 action *in vivo* based on the lack of association between circulating sTie2 and disease severity in septic and non-septic critically ill patients^34^.

In contrast to natural circulating sTie2, a ligand trap consisting of a dimeric Tie2 ectodomain fusion protein was far more effective at blocking Angpt1-induced cellular Tie2 activation. This likely reflects the increased avidity of the dimeric trap providing it with a significant kinetic advantage relative to monomeric sTie2.

In conclusion, this study shows that normal and pathological levels of sTie2 have little effect on Angpt1-induced Tie2 clustering and activation in endothelial cells. These data suggest that therapeutic targeting sTie2 in conditions where the ectodomain is elevated would have little if any effect. However, dimeric sTie2 is able to inhibit Angpt1 activation of endothelial Tie2 at much lower concentrations. Thus in contrast to endogenous monomeric sTie2, the dimeric engineered ectodomain could provide a route for suppression of angiopoietin action.

## Methods

### Materials

Recombinant human Angpt1, antibodies recognizing Tie2 extracellular domain and anti-phosphoY^992^-Tie2 antibody were obtained from R&D Systems. Human umbilical vein endothelial cells (HUVEC) were maintained in Medium 200 supplemented with Low Serum Growth Supplement and 10% (v/v) foetal bovine serum. Monomeric sTie2 and dimeric sTie2 were purified from medium of transfected HEK-293 cells using the transfection and purification methods we previously described for dimeric sTie2^35^. For monomeric sTie2, cDNA encoding Tie2 ectodomain residues 1–442 (including the secretory leader sequence) was obtained as previously described^35^ and cloned into the expression vector in frame with a C-terminal hexahistidine tag. cDNA encoding dimeric sTie2 was as described previously^35^. Following purification protein was dialysed against phosphate buffered saline (PBS) with 10% glycerol and protein concentration determined. Purity was assessed by sodium dodecyl sulphate polyacrylamide gel electrophoresis (SDS-PAGE) and Coomassie staining and was routinely better than 99%. All other materials were as previously described^36^.

### Mathematical model and numerical simulations

The mathematical model based on biochemical kinetics^20^ for Angpt1-Tie2 binding and corresponding numerical simulations were performed with Matlab (R2015b) SimBiology package and described in the Supplementary Information.

### Quantification of cellular Tie2

The number of Tie2 receptors in HUVEC was determined by immunoblotting. Cells were counted and then lysed in Laemmli sample buffer containing 100 mM dithiothreitol Cleared lysates were heated to 100 °C for 5 min before resolving proteins by SDS-PAGE. In parallel known amounts of recombinant Tie2 were also resolved by SDS-PAGE. Proteins were transferred to PVDF or nitrocellulose membranes and probed with Tie2 antibody. Immunoreactive proteins were detected with peroxidase conjugated secondary antibody and chemiluminescent detection^37^. Western blots were quantified by densitometric analysis of images. Cellular Tie2 was quantified by comparison of band density with the standard curve constructed from known amounts of recombinant Tie2 blotted and probed on the same membranes. The number of Tie2 molecules at the cell surface was derived from the percentage of total cellular Tie2 that is surface localized and total number of Tie2 receptors in each cell. The percentage of total Tie2 at the cell surface was determined by surface biotinylation as we previously described^38^ and quantification of biotinylated and non-biotinylated Tie2 by immunoblotting following separation with streptavidin-agarose.

### Cellular Tie2 activation

Prior to ligand treatment HUVEC were incubated in serum-free medium for one hour. As appropriate, and indicated in Results, cells were treated with 0.18 nM Angpt1 in the absence and presence of sTie2 for 15 min. In preliminary experiments we confirmed that at 15 min Angpt1-induced Tie2 phosphorylation had plateaued Supplementary Fig. S3). Cells were then rapidly placed on ice, medium aspirated and cells lysed by addition of Laemmli sample buffer containing 100 mM dithiothreitol and cell scraping. Cleared lysates were heated to 100 °C for 5 min before resolving proteins by SDS-PAGE. Proteins were transferred to PVDF or nitrocellulose membranes and probed with anti-phosphoY^992^-Tie2 antibody. Immunoreactive proteins were detected with peroxidase conjugated secondary antibody and chemiluminescent detection^37^. Blots were stripped and re-probed with anti-Tie2 or anti-vinculin to assess loading. Bands on Western blots were quantified by densitometric analysis.

## Electronic supplementary material

Supplementary Information
