# Supplementary material for: Regulation of Angiopoietin Signalling by Soluble Tie2 Ectodomain and Engineered Ligand Trap
Source: Sci Rep. 2017 Jun 16;7:3658. doi: 10.1038/s41598-017-03981-6 (PMC5473842; doi:10.1038/s41598-017-03981-6)
Supplement: Supplementary file 1 — Supplementary Information [file 41598_2017_3981_MOESM1_ESM.pdf]

Regulation of Angiopoietin Signalling by Soluble Tie2 Ectodomain and Engineered Ligand Trap

Deborah O.A. Alawo, Tariq A Tahir, Marlies Fischer, Declan G. Bates, Svetlana R Amirova and Nicholas PJ Brindle

Supplementary Information

A simple mathematical model of biochemical kinetic interactions for Ang1 interaction with soluble Tie2 and cell surface Tie2 is illustrated in Fig. 1a and was implemented in Matlab (R2015b) SimBiology software (Mathworks). The interaction of tetrameric Ang1 with sTie2 was modelled as a series of binding steps depicted below:

$$\mathrm{A4} \quad + \quad \mathrm{S} \quad \underset{k_{-1}}{\overset{k_1}{\rightleftharpoons}} \quad \mathrm{A3S}$$

$$\mathrm{A3S} \quad + \quad \mathrm{S} \quad \underset{k_{-1}}{\overset{k_1}{\rightleftharpoons}} \quad \mathrm{A2S2}$$

$$\mathrm{A2S2} \quad + \quad \mathrm{S} \quad \underset{k_{-1}}{\overset{k_1}{\rightleftharpoons}} \quad \mathrm{A1S3}$$

$$\mathrm{A1S3} \quad + \quad \mathrm{S} \quad \underset{k_{-1}}{\overset{k_1}{\rightleftharpoons}} \quad \mathrm{AS4}$$

where A denotes angiopoietin with suffix indicating the number of free binding sites, and S denotes soluble Tie2 with the suffix indicating the number of S bound.

Binding of tetrameric Ang1 to membrane localized Tie2 is depicted as an initial recruitment of Ang1 from the extracellular space to membrane-localized Tie2:

$$\mathrm{A4} \quad + \quad \mathrm{M} \quad \underset{k_{-1}}{\overset{k_1}{\rightleftharpoons}} \quad \mathrm{A3M}$$

followed by binding of additional membrane-localized Tie2 receptors to the complex:

$$A3M + M \underset{k_{-1}}{\overset{k_2}{\rightleftarrows}} A2M2$$

$$A2M2 + M \underset{k_{-1}}{\overset{k_2}{\rightleftarrows}} A1M3$$

$$A1M3 + M \underset{k_{-1}}{\overset{k_2}{\rightleftarrows}} AM_4$$

where M denotes cell membrane Tie2 with the suffix indicating the number of M bound.

Importantly, once bound to membrane-localized Tie2 (Eq. 5 below) the ligand accesses cellular Tie2 receptors in the more restricted compartment circumscribed by the accessible cellular surface area and the reach of the ligand from the surface (Fig. 1b).

These interactions were implemented in a series of ordinary differential equations according to laws of mass action kinetics as indicated below:

$$\frac{d[A3S]}{dt} = k_1[A4]*4*[S] - k_{-1}*[A3S] \qquad \text{(Eq. 1)}$$

$$\frac{d[A2S2]}{dt} = k_1[A3S]*3*[S] - k_{-1}*2*[A2S2] \qquad \text{(Eq. 2)}$$

$$\frac{d[A1S3]}{dt} = k_1[A2S2]*2*[S] - k_{-1}*3*[A1S3] \qquad \text{(Eq. 3)}$$

$$\frac{d[AS4]}{dt} = k_1[A1S3]*[S] - k_{-1}*4*[AS4] \qquad \text{(Eq. 4)}$$

$$\frac{d[A3M]}{dt} = k_1[A4]*4*[M] - k_{-1}*[A3M] \qquad \text{(Eq. 5)}$$

$$\frac{d[A2M2]}{dt} = k_2[A3M]*3*[M] - k_{-1}*2*[A2M2] \quad \text{(Eq. 6)}$$

$$\frac{d[A1M3]}{dt} = k_2[A2M2]*2*[M] - k_{-1}*3*[A1M3] \quad \text{(Eq. 7)}$$

$$\frac{d[AM4]}{dt} = k_2[A1M3]*[M] - k_{-1}*3*[AM4] \quad \text{(Eq. 8)}$$

Parameters used in mathematical model

Compartment volumes

Our model represents a typical cell culture experiment of a $10cm^2$ tissue culture dish containing $4 * 10^5$ cells and a medium volume of 1 ml. Interactions with sTie2 occur in the extracellular medium whereas once recruited to Tie2 at the cell membrane further interactions of ligand:cellular Tie2 occur in the cell membrane compartment (Fig. 1b). Once bound to the cell surface the effective volume accessible by the ligand is that of the cell surface area multiplied by the height above the surface that can be sampled by the ligand. Assuming a flexible ligand this height is illustrated in Figure 1c and is 25nm for tetrameric Ang1 based on the 21 amino acid linker (approximately 0.35nm/residue[1]) between the ligand binding domain and oligomerization domain. The length of distance between the end of the linker and receptor binding interface is 4.5nm as determined from the structure of the Ang1 FReD[2] (Fig. 1c). The volume of this membrane compartment is therefore $2.4885x10^{-8}$ l.

Tie2

Cellular Tie2 was quantified as described in Materials and Methods. This yielded 179,880 +/- 12, 202 (mean +/- SEM, n=5 experiments) receptors per endothelial cell and 86.9+/- 2.8 % (mean +/- SEM, n=3 experiments) of this is on the cell surface. Each $10cm^2$ dish contained $4x10^5$ cells.

Kinetic rate constants

k_1 and k_2 are the forward rate constants for binding of the monomeric receptor binding domain of ligand with monomeric receptor ectodomain in the extracellular and membrane compartments respectively. Implementation of the two-compartment model in SimBiology requires use of compartment volumes and moles of reactants. Therefore k_1 and k_2 were derived from the measured molar forward rate constant ($2.7 * 10^6$ M^{-1} s^{-1}) for this monomer:monomer interaction[3] adjusted to account for the extracellular and membrane compartment volumes respectively.

At the endothelial cell surface Tie2 has been shown to exist mainly as groups of two receptors in close proximity[4] (less than 1.4nm apart). Therefore once the ligand has bound its first surface receptor it is likely to encounter a second

receptor in the search space of the extended ligand (Fig. 1b). However, to bind additional receptors the complex may need to travel within the membrane. The association rate for these subsequent binding events are therefore likely to be lower than in the extracellular space as movement of the ligand:receptor complex and free receptor will be impeded in two dimensions by the viscosity of the membrane. Typical lateral diffusion coefficients of proteins in solution are in the region of 10^{-6} to $5 * 10^{-7}$ cm^2 s^{-1} whereas $5 * 10^{-9}$ to 10^{-10} cm^2 s^{-1} is typical of proteins in membranes[5,6]. To account for this we decreased the k2 forward constant for the third and fourth binding event by the ratio of lateral diffusion coefficient in membrane:solution, 10^{-4}.

The reverse rate constant, k_{-1}, was that measured for dissociation of monomeric ligand from monomeric receptor[3]. Surface plasmon resonance revealed two stage dissociation of Ang1 monomer from Tie2 ectodomain, an initial rapid dissociation phase (0.748 s^{-1}) followed by a slower phase (0.16 s^{-1})[3]. In this model the 0.748 s¯1 rate constant was used as this is the constant for initial disengagement of ligand from receptor.

Parameter	Definition	Starting Value
V_e	Extracellular volume	$1 * 10^{-3}$ l
V_m	Membrane compartment volume	$2.4885 * 10^{-8}$ l
M	Total cellular Tie2	$1.03934 * 10^{-13}$ moles
k_1	Forward rate constant in extracellular medium	$2.7 * 10^{9}$ $mole^{-1}$ s^{-1}
k_2	Forward rate constant in membrane compartment	$1.08499 * 10^{14}$ $mole^{-1}$ s^{-1}
k_{-1}	Reverse rate constant	0.748 s^{-1}

Table S1: Summary of parameters used in model

References

s1. Huston, J. S., Levinson, D., Mudgett-Hunter, M., Tai, M.-S., Novotnỳ, J. v., Margolies, M. N., Ridge, R. J., Bruccoleri, R. E., Haber, E. & Crea, R. Protein engineering of antibody binding sites: Recovery of specific activity in an anti-digoxin single-chain fv analogue produced in escherichia coli. *Proceedings of the National Academy of Sciences* **85**, 5879-5883 (1988).

s2. Barton, W. A., Tzvetkova-Robev, D., Miranda, E. P., Kolev, M. V., Rajashankar, K. R., Himanen, J. P. & Nikolov, D. B. Crystal structures of the tie2 receptor ectodomain and the angiopoietin-2-tie2 complex. *Nat. Struct. Mol. Biol.* **13**, 524-532 (2006).

s3. Yeykal, C. C. *Structural and biochemical characterization of angiopoietin-1* PhD thesis, The University of Chicago, (2012).

s4. Bogdanovic, E., Coombs, N. & Dumont, D. J. Oligomerized tie2 localizes to clathrin-coated pits in response to angiopoietin-1. *Histochem. Cell Biol.* **132**, 225-237 (2009).
s5. Raj, T. & Flygare, W. H. Diffusion studies of bovine serum albumin by quasielastic light scattering. *Biochemistry* **13**, 3336-3340 (1974).
s6. Liebman, P. A. & Entine, G. Lateral diffusion of visual pigment in photoreceptor disk membranes. *Science* **185**, 457-459 (1974).

Supplementary Figures

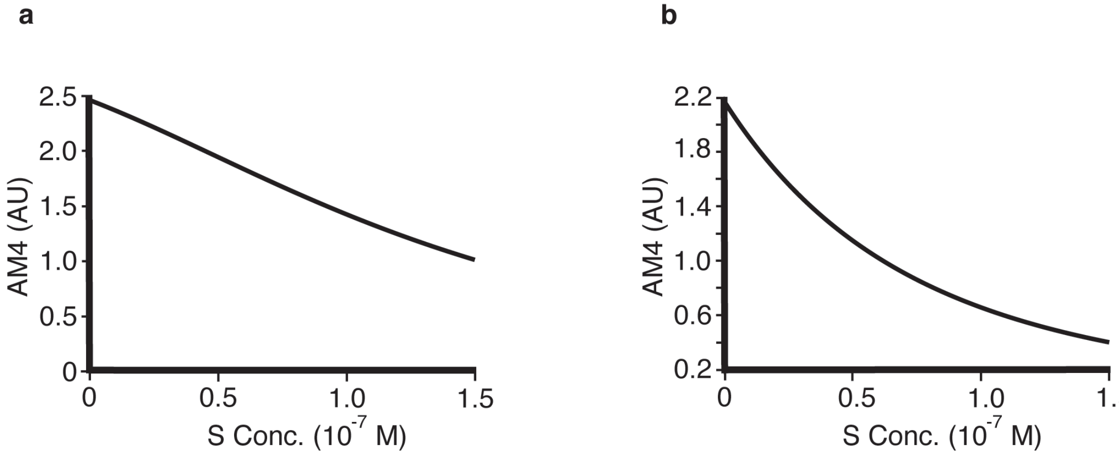

Figure S1. Numerical simulations of the effects of sTie2 on AM4 at different levels of cellular Tie2. (**a**) Concentration-dependence of sTie2 effects on formation of signalling-competent AM4 complex at the cell surface in cells with ten-fold higher cellular Tie2 than simulations shown in Figures 1-3. The absolute level of AM4 in the absence of sTie2 is $1.6 * 10^3$ times higher than the corresponding level of AM4 in Figure 3f (**b**) Concentration-dependence of sTie2 effects on formation of signalling-competent AM4 complex at the cell surface in cells with ten-fold lower cellular Tie2 than simulations shown in Figures 1-3. The absolute level of AM4 in the absence of sTie2 is $7 * 10^4$ times lower than the corresponding level of AM4 in Figure 3f.

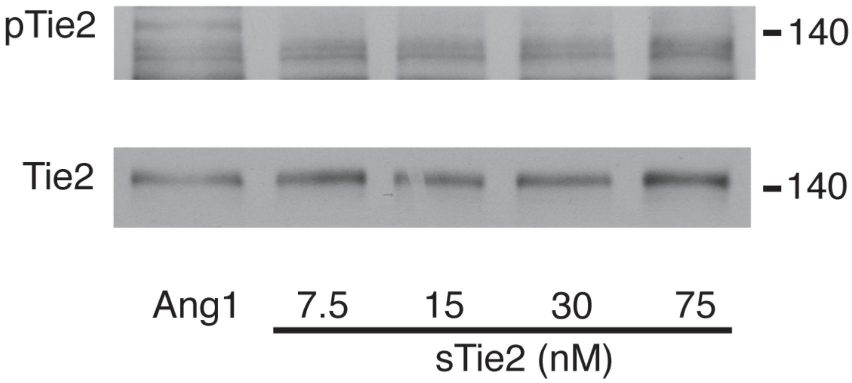

Figure S2. Effects of sTie2 on activation of cell surface Tie2. HUVEC were stimulated with 0.18nM Ang1 or monomeric sTie2 at the concentrations shown for 15 min. Cells were lysed and activated cellular Tie2 detected with anti-phosphoY^{992}-Tie2 immunoblotting (upper panel). Blots were stripped and probed with anti-Tie2 (lower panel). The positions of phosphorylated Tie2 (pTie2) and total Tie2 (Tie2) are indicated. The position of the 140kDa marker is also shown. Blots have been cropped for clarity.

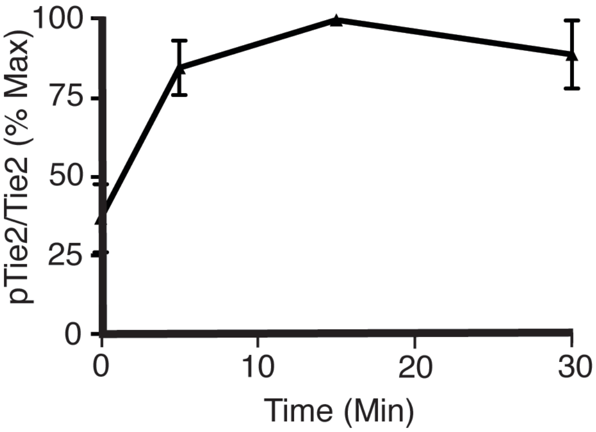

Figure S3. Time course of Angpt1 activation of cellular Tie2 phosphorylation in HUVEC. HUVEC were stimulated with 0.18nM Angpt1 for the times indicated. Cells were lysed and activated cellular Tie2 detected with anti-phosphoY^{992}-Tie2 immunoblotting. Blots were stripped and probed with anti-Tie2 to quantify loading of tracks. Blots from four independent experiments assessing cellular Tie2 activation were quantified by densitometric scanning and presented as mean and SEM.

Supplementary Information - Full-length blots

Images of the full-length blots used in Figures 1, 4 and S2 are shown below

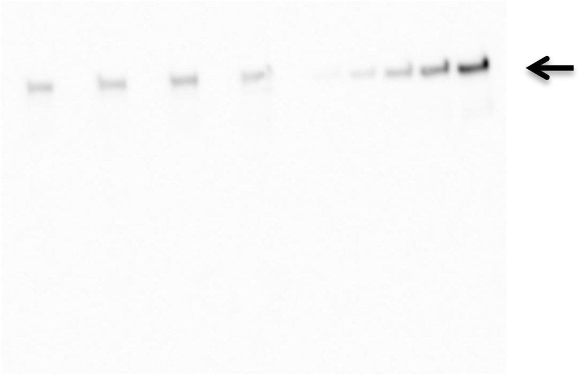

Tie2 probe

Full-length blot from Figure 1d Tie2 quantification. The position of Tie2 is indicated with an arrow.

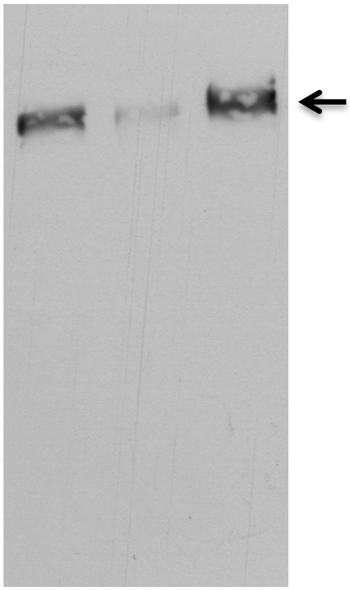

Tie2 probe

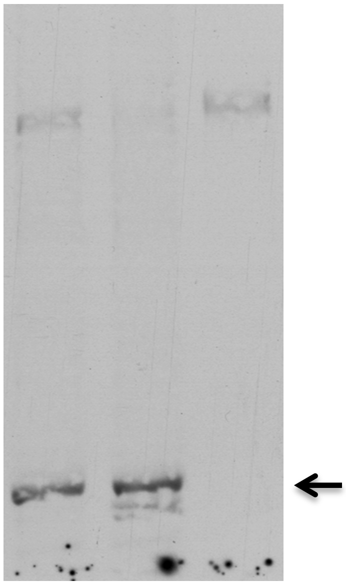

Tubulin probe

Full-length blot from Figure 1d. The positions of Tie2 and tubulin are indicated with arrows

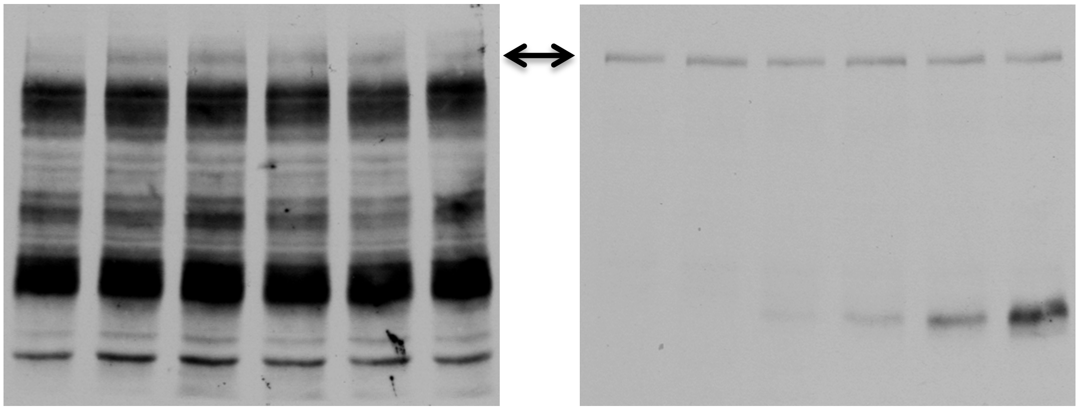

pTie2 probe Tie2 probe

Full-length blot from Figure 4a. The positions of pTie2 and Tie2 are indicated by the double-headed arrow.

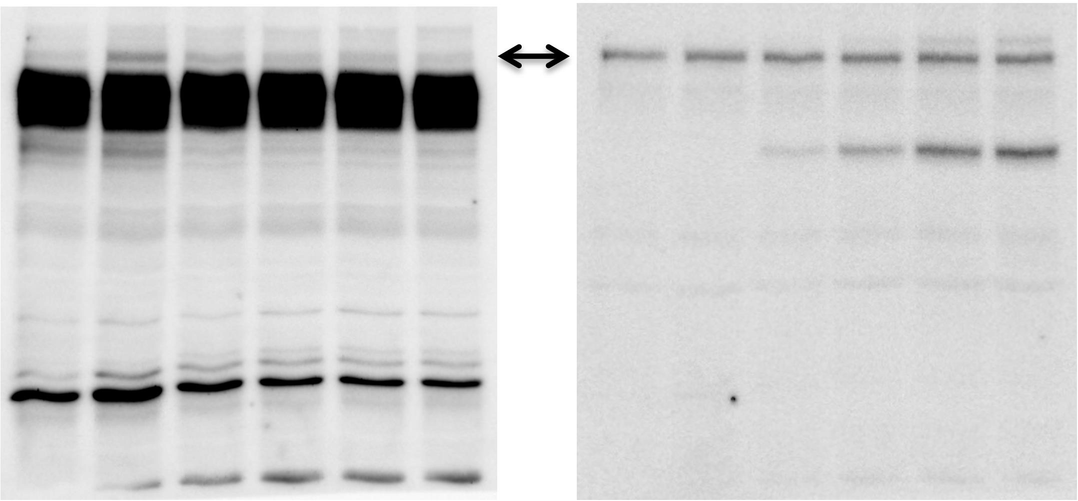

pTie2 probe Tie2 Probe

Full-length blot from Figure 4b. The positions of pTie2 and Tie2 are indicated by the double-headed arrow.

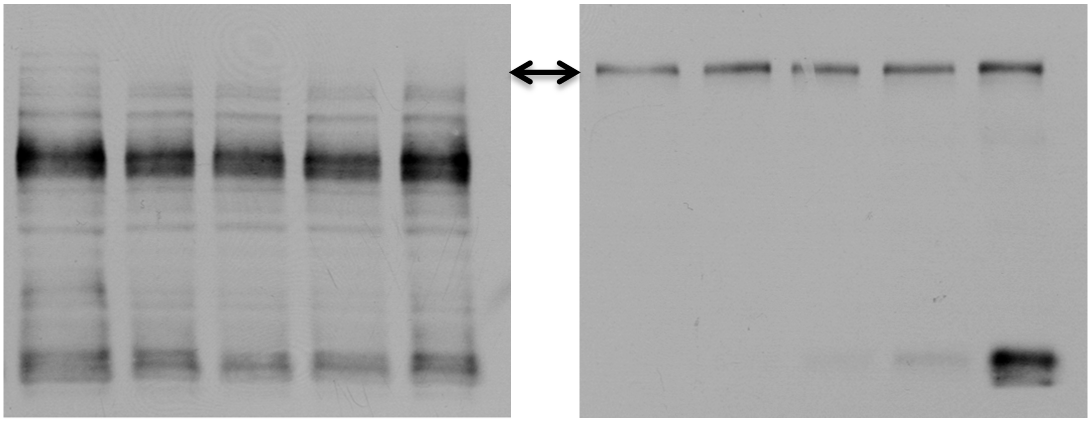

pTie2 probe

Tie2 probe

Full-length blot from Supplementary Figure S2. The positions of pTie2 and Tie2 are indicated by the double-headed arrow.
